# Surface energy of nanoparticles – influence of particle size and structure

**DOI:** 10.3762/bjnano.9.211

**Published:** 2018-08-23

**Authors:** Dieter Vollath, Franz Dieter Fischer, David Holec

**Affiliations:** 1 NanoConsulting, Primelweg 3, D-76297 Stutensee, Germany; 2 Institute of Mechanics, Montanuniversität Leoben, A-8700 Leoben, Austria; 3 Department of Physical Metallurgy and Materials Testing, Montanuniversität Leoben, A-8700 Leoben, Austria

**Keywords:** ab initio calculations, classical thermodynamics, molecular dynamics simulation, surface energy, surface layer

## Abstract

The surface energy, particularly for nanoparticles, is one of the most important quantities in understanding the thermodynamics of particles. Therefore, it is astonishing that there is still great uncertainty about its value. The uncertainty increases if one questions its dependence on particle size. Different approaches, such as classical thermodynamics calculations, molecular dynamics simulations, and ab initio calculations, exist to predict this quantity. Generally, considerations based on classical thermodynamics lead to the prediction of decreasing values of the surface energy with decreasing particle size. This phenomenon is caused by the reduced number of next neighbors of surface atoms with decreasing particle size, a phenomenon that is partly compensated by the reduction of the binding energy between the atoms with decreasing particle size. Furthermore, this compensating effect may be expected by the formation of a disordered or quasi-liquid layer at the surface. The atomistic approach, based either on molecular dynamics simulations or ab initio calculations, generally leads to values with an opposite tendency. However, it is shown that this result is based on an insufficient definition of the particle size. A more realistic definition of the particle size is possible only by a detailed analysis of the electronic structure obtained from initio calculations. Except for minor variations caused by changes in the structure, only a minor dependence of the surface energy on the particle size is found. The main conclusion of this work is that surface energy values for the equivalent bulk materials should be used if detailed data for nanoparticles are not available.

## Review

### Introduction

With respect to the thermodynamics of small particles, the surface energy is an essential, and in many cases the dominant, quantity. Therefore, it is astonishing that so much uncertainty remains about this physical quantity. The concept of surface energy was introduced by Gibbs using the term “surface tension” [1–2]. In the meantime, it has become clear that in case of solids one has to distinguish between “surface energy” and “surface stress”. Both quantities are related by the Shuttleworth equation [3]. Since the surface stress, σ, exerts a pressure, *p*, on a curved solid (e.g., for a sphere with the radius *R*, *p* = 2σ/*R*) the elastic strain energy is stored in the particle. The surface energy may be defined as the as excess energy, i.e., the difference in the energy between a particle and the same number of atoms in an infinitely extended solid [4]. This definition does not take into account that the surface energy may be different for different crystallographic planes at the surface of a facetted particle. It is common that only average values are discussed, as is the case in this paper.

According to Fried and Gurtin [5], the surface energy, γ, may be split-up into a term depending on the binding in the solid (“chemical contribution”), γ_bond_, and into a minor term caused by the stress state due to the surface stress (“mechanical contribution”), γ_mech_, as

[1]
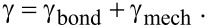


The contribution of the mechanical energy to the surface energy is, compared to the chemical contribution, minor [3]. Based on this clarification, it is obvious that the lattice contraction data for small particles, abundant in the literature, may be used to calculate the size dependent surface stress, (see, e.g., [6–8]) but not, as it is done sometimes [9–10], to calculate the surface energy (“surface tension”).

**Figure 1:**
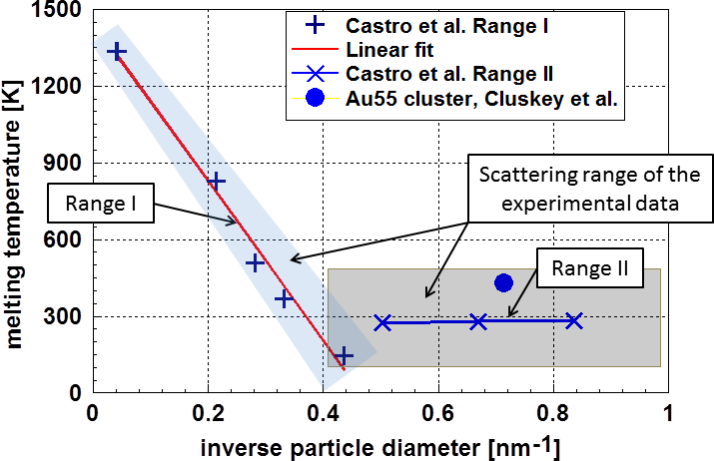
Melting temperature of gold nanoparticles according to Castro et al. [11] and Cluskey et al. [13]. One realizes two ranges in the data of Castro et al.: Within the experimental error bar (shaded area) results of Castro et al., the result of Cluskey et al., obtained on Au_55_ clusters, is also located.

The analysis of the surface energy data of nanoparticles and the dependence of the surface energy on particle size and temperature is of essential importance because the phase (crystalline or liquid) depends on the particle size. Additionally, it is possible that the particles form a stable glass phase with very different properties. A typical example is demonstrated in Figure 1, which depicts the course of the melting temperature of gold particles on the inverse particle size [11]. Besides the well-known inverse linear dependence according to Pawlow [12] (Range I), *T*_melt–bulk_ − *T*_melt–nano_ 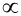 γ/*d*, a range where the melting temperature is independent of the particle size (Range II) can also be observed. The experimental error bar also covers the results of other authors. In this range, the particles are glassy, indicating a structure more stable than a crystalline one [13–14].

The applicability of the Pawlow-relationship over a wide range of particle sizes, as shown in Figure 1, is a strong indicator that the surface energy of gold particles is, down to a particle diameter, *d* of ≈2 nm, largely independent of the particle size. Other experimental results on aluminum [15] and lead [16] indicate the correctness of this finding. Since a direct measurement of the surface energy of nanoparticles is more or less impossible, many attempts have been made to calculate this quantity. It is astonishing and disconcerting that calculations based either on classical thermodynamics or on molecular dynamics and ab initio methods have generally resulted in an opposite tendency with respect to the influence of the particle size. Obviously, there are additional structural phenomena not taken into account. It is the intention of this paper to depict these differences and to offer some explanations. To do this, the basic ideas of both approaches are explained in this paper.

### Approach based on classical thermodynamics and continuum considerations

When Gibbs [1–2] introduced surface energy using the term surface tension he predicted a decrease of the surface energy with decreasing droplet size. As an early approach, analyzing the equilibrium between a liquid phase and the surrounding vapor, Tolman [17] estimated the surface energy of small droplets. These considerations led to the relation

[2]
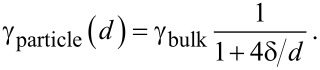


In Equation 2 the quantity *d*, where *d* = 2*R*, stands for the particle diameter and *R* for its radius; δ is a material-dependent constant called the Tolman-length, which is in the range of 10^−10^ m. Tolman stated that his approach may fail in the case of very small particles because the atomic structure of the particle is not taken into account. Furthermore, he stated that the quantity δ is not necessarily constant; it could even change its sign under certain conditions. It is obvious from Equation 2 that, in case of δ > 0, the surface energy is reduced with decreasing particle diameter.

More generally, one may assume that the surface energy is proportional to the number of broken bonds at the surface atoms times the energy per bond. Therefore, one may expect a relation such as

[3]
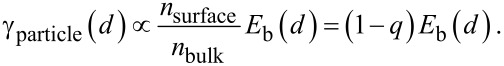


In Equation 3 the quantity *E*_b_(*d*) stands for the energy per bond and the quantity *q,* the relative coordination number, stands for the ratio of the coordination number of a surface atom *n*_surface_ to that of an atom in the undisturbed bulk material *n*_bulk_. Certainly, the validity of the Tolman equation (Equation 2) requires a strong dependence of the binding energy *E*_b_(*d*) with the particle size.

**Figure 2:**
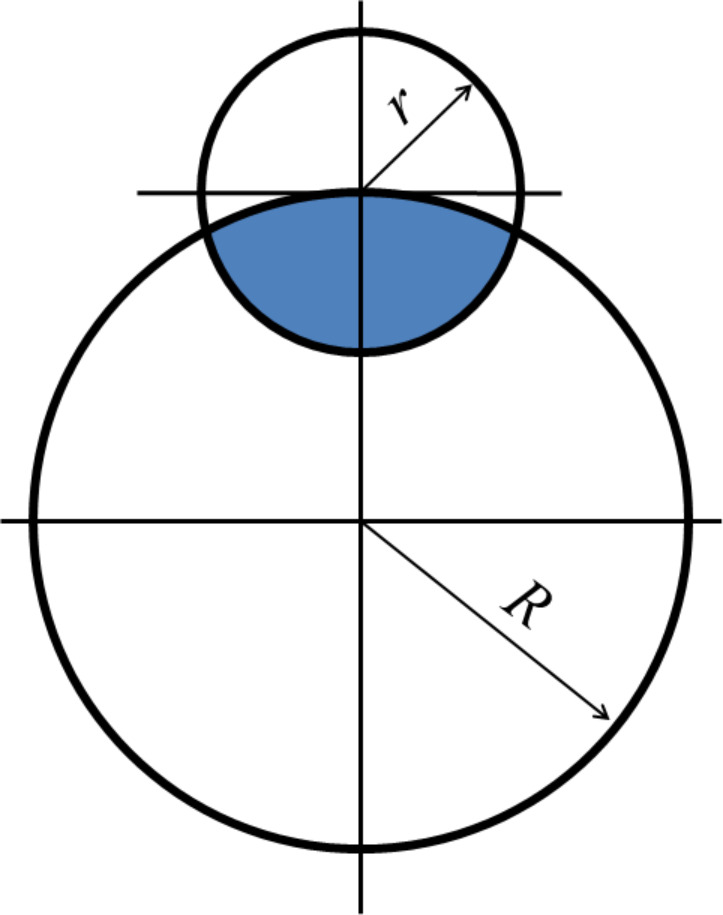
Visualization of the setting for the estimation the number of next neighbors (coordination number) of an atom at the surface of a spherical particle with diameter *d* = 2*R*. The volume of the intersection of this sphere with a second sphere of the radius *r,* representing the distance to the next neighbors, allows an approximation for the possible number of next neighbors.

Models using the ratio *q* yield in the same tendency as Tolman introduced. This ratio *q* is also used to estimate the size dependence of the thermodynamic quantities [18–22]. Similar estimations, using the number of broken bonds at the surface, were performed by Lu and Jiang [23]. The result of counting broken bonds or applying relative coordination numbers depends on the crystal structure and on the crystallographic plane. Therefore, quite often, one may find examples of these coordination numbers for different structures. Consequently, such a model is not suitable for generalized considerations. For the surface coordination number, the same crystallographic aspects are valid as in case of the broken bonds at the surface. For these more generalized considerations, it is sufficient to apply a simple first approximation. This allows for estimating the relative number of neighbors of an atom located at the surface of a spherical particle. To do this, a particle with the diameter *d* = 2*R* and the distance *r* to next neighbors is considered. In this case, the number of next neighbors is proportional the volume of the intersection between the particle and a sphere with the diameter 2*r* having its center at the surface of the particle. (Certainly, this simplified treatment is not expedient for anisotropic materials.) These considerations are visualized in Figure 2.

From geometric considerations, as depicted in Figure 2, the relative coordination number *q*, the ratio of the coordination number of an atom at the surface of the particle to the coordination number of an atom in the bulk – is calculated as ratio of the volumes yielding

[4]
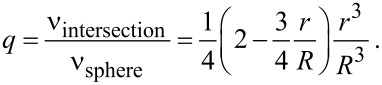


This equation may be approximated with sufficient accuracy according to [24] as

[5]
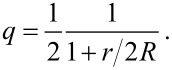


**Figure 3:**
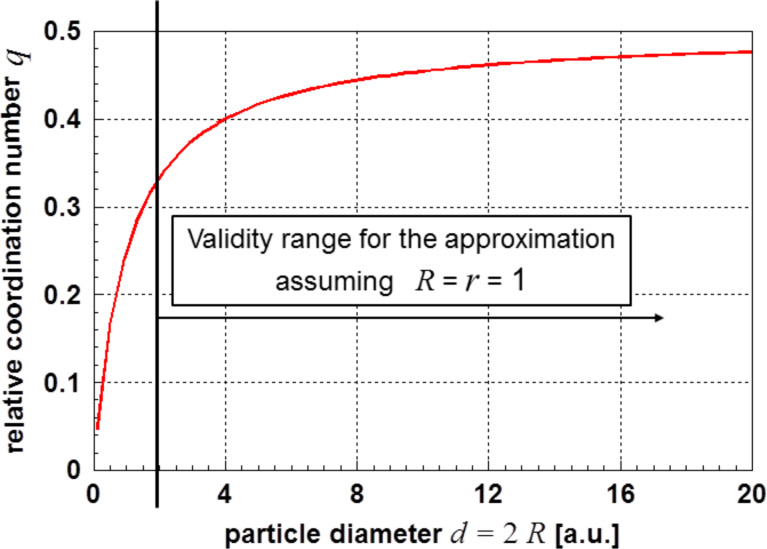
Relative coordination number *q* of an atom at the surface of a spherical particle as function of the particle diameter *d* = 2*R* using Equation 5.

Comparing Equation 2 and Equation 5 with respect to the functional relations for the dependency on *d* = 2*R*, one realizes that these two expressions are, except for the factor 0.5, more or less identical. Both equations use an empirical parameter (*r* or δ) as a variable. Equation 4 is just the exact expression of this approach. Looking at Figure 2, it is obvious that the smallest particle radius where above considerations are valid is *R* = *r*. Analyzing Equation 5, one realizes that the quantity *q* is 0.5 at a plane surface (*R* → ∞) and 1/3 for *R* = *r*. The quantity *q*, according to Equation 5, as a function of the particle diameter is plotted in Figure 3.

For particle sizes smaller than *R* = *r* the range of next neighbors of diametrically opposite surface atoms overlap; this is the limit of this model. Therefore, from Figure 3 one learns that the quantity *q* gives useful values *q* ≥ 1/3 only in the size range for particle diameters larger than 2*r.* Possibly, this type of functional relationship leads to a description of the reality.

As shown in Equation 3, the surface energy is proportional to the relative coordination number. Theoretically more elaborated studies lead to descriptions of the type [17,19–20] as

[6]
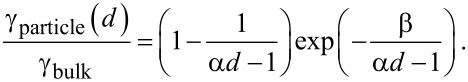


**Figure 4:**
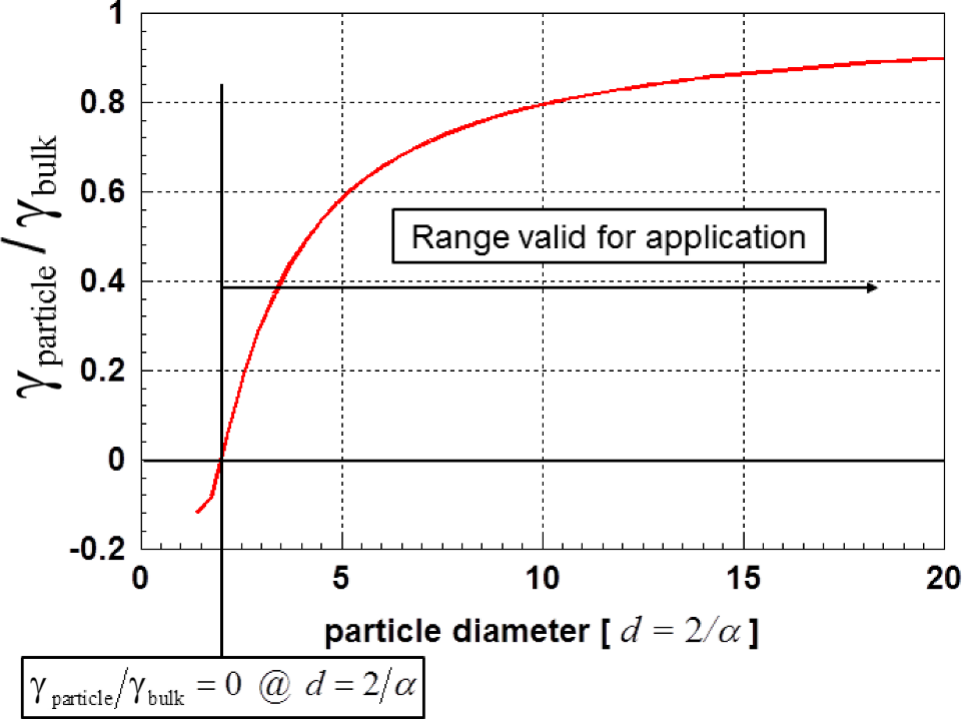
Graph of Equation 6. Also in this case, a lower limit for the particle diameter exists (α = β = 1).

In Equation 6 the quantities α and β are particle-size-independent constants. The quantity of these constants depends on the applied theory (see, e.g., [23,25–26]). For *d* = 2/α Equation 6 delivers a value of zero for the surface energy; for values *d* < 2/α the results are negative. Approximations of the surface energy are only possible in the range *d* > 2/α. Figure 4 displays the course of the surface energy as described by Equation 6. The limitations for meaningful results are well visible in this graph.

**Figure 5:**
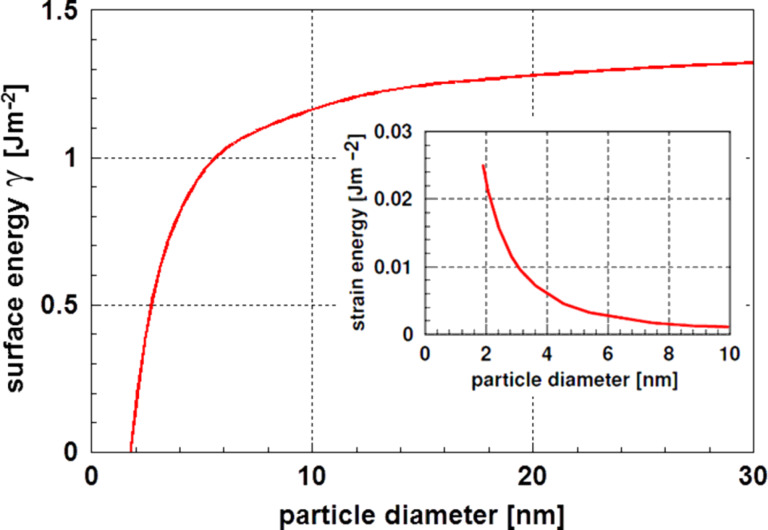
Surface energy for gold nanoparticles as function of the particle diameter according to Gang et al. [26]. The insert shows the specific strain energy related to the particle surface. Excerpt with permission from [26], copyright 2006 by the American Physical Society.

As a further example for theoretically derived values for the surface energy γ, Figure 5 displays the surface energy of gold nanoparticles as a function of the particle diameter [26]. These values were obtained using a function of the type of Equation 6. As was discussed in context with Equation 6, these results are only valid for γ ≥ 0. To demonstrate the insignificant role of the specific surface strain energy on the surface energy, the insert in Figure 5 displays the particle size dependence of this quantity. Although its contribution increases drastically with decreasing particle size, its influence is negligible.

More recent, Xie et al. [27] published a model to estimate the surface energy for nanoparticles, which leads to a very high constant value of the surface energy for small particle diameters. However, this result was obtained by assuming the ratio of the number of bonds in the interior is 0.25. This ratio is equivalent to number of bonds in the bulk to the number of bonds at the surface. The quantity *q* is defined in Equation 4 or Equation 5 and set as *q* = 0.25. Additionally, the energy for one bond, *E*_b_(*d*), was set constant and is not particle size dependent. However, the bonding energy in-between the atoms *E*_b_(*d*) decreases significantly with increasing particle size [28]. Models have been published showing that the energy *E*_b_(*d*) is (in good approximation) inversely proportional to the particle size for small particles [28], leaving the concept of excess energy for the surface energy. This approach was published in a series of papers discussing the particle size dependence of properties. In a communication on scaling laws of physical properties, Guibiers et al. [29–30] showed that many of the structural properties of small particles show an inverse proportionality with particle size. This led to the development of a general law governing most of the properties. The size dependence of the property ξ is given as

[7]
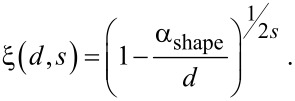


In Equation 7 the quantity *d* stands for the particle diameter, α_shape_ is a shape-dependent factor, *s* = 0.5 for properties related to quasi-particles following the Fermi–Dirac statistics (e.g., melting, ferromagnetism, diffusion, etc.) and *s* = 1 for properties related to quasi-particles following the Bose–Einstein statistics (e.g., superconductivity or vibration) [30–32]. Karasevskii [33] started with a different approach based on size dependent quantization of vibrational modes, resulting in an inverse proportionality between thermodynamic quantities and particle size.

**Figure 6:**
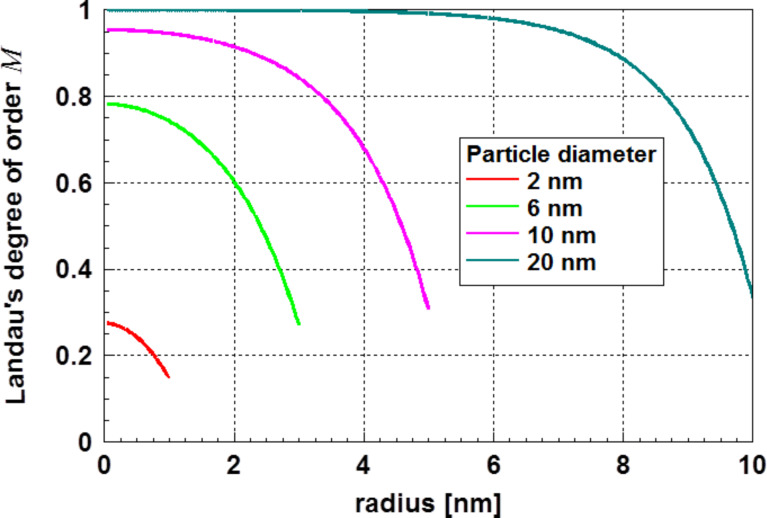
Landau’s order parameter *M* for tin particles of different particle diameters as function of the radius at a temperature of 440 K. The term “radius” represents the radial distance to the particle center [35].

The considerations above assume implicitly that the bonding energy in between the atoms of the particle and at the surface is independent of the particle size. This assumption is not necessarily correct [25]. In this context, the melting processes of nanoparticles must be considered. The general assumption of homogenous melting, which means that the whole particle melts suddenly at the melting point, is not realistic in many cases. More realistic assumptions are either the formation of a liquid or liquid-like layer at temperatures significantly below the melting temperature or a continuous process of melting starting at the surface. Thermodynamic calculations support these models [34–35]. Sakai [34] concludes that the phenomenon of a liquid-like surface layer is, at least for the example of lead, restricted to particles larger than a critical size in the range of 4 nm; otherwise, the particles melt all at once. For larger particles, this layer may reach a thickness of more than 1.2 nm. For smaller particles such a layer may be extremely thin. The results published by Chang and Johnson [35] do not show such a size limit. In contrast, the phenomenon of premelting at the surface enhances with decreasing particle size. Figure 6 displays Landau’s order parameter *M* [36] for different particle diameters as function of the radial distance from the particle center, denoted as radius [35]. This order parameter obtains the value 1 for perfectly crystallized and 0 for liquid material.

Certainly, such a surface layer with reduced crystalline order has a surface energy, which is closer to that of a liquid rather than a crystalline solid. Also this result supports arguments leading to the decrease of the surface energy with decreasing particle size, at least in the vicinity of the melting temperature. Certainly, this behavior influences the surface energy.

Phase field studies by Levitas and Samani [37–38] analyzed melting and solidification of small particles. In general, these studies led to very similar conclusions as reported by Chang and Johnson [35]. However, it must be noted that Levitas and Samani found that in addition to the traditionally described barrierless melting, a hysteretic jump-like premelting phenomenon, also connected to bi-stable states between solid and liquid phases occurs. Also these results predict (for certain particle sizes) the existence of a layer with reduced order, possibly a premelted liquid layer at the surface of a crystallized particle.

**Figure 7:**
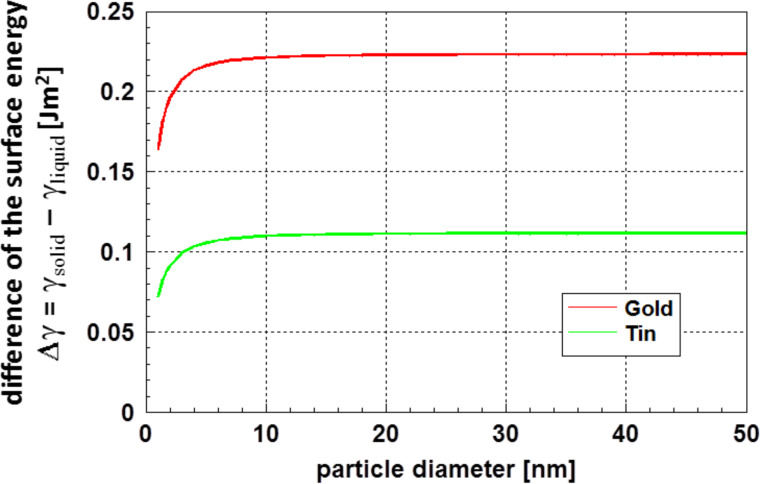
Difference of the surface energy between the solid and the liquid state at the melting temperature of the particles as function of the particle diameter for gold and tin [24].

From the experimental data on the melting of gold [11,39] and tin [40–41] particles, Vollath and Fischer [24] calculated the difference between the surface energy of solid and liquid nanoparticles, Δγ = γ_solid_ – γ_liquid_, as a function of the particle diameter. As shown in Figure 7, the results of these calculations indicated a reduction of this difference for small particles with decreasing particle diameter.

This reduction of the surface energy shown in Figure 7 is certainly connected to the formation of a disordered surface layer, as it was shown by Chang and Johnson [35]. For small particles, Figure 6 and Figure 7 clearly demonstrate (with a high probability) that at least the surface of nanoparticles loses order and becomes liquid-like. Therefore, as γ_liquid_ < γ_solid_ is always valid, an increase of the surface energy with decreasing particle size cannot be expected.

### Approach to the surface energy from atomistic calculations

#### Molecular dynamics simulations

Molecular dynamics simulations describe the spatial movement of atoms in a solid. The atoms interact and move in a given time frame due to the action of interatomic forces. The mathematical description of this potential is one of the critical problems of this type of simulation [42]. In general, molecular dynamic simulations are based on the laws of classical Newtonian mechanics of multibody systems. Later developments apply potentials derived from quantum-mechanical laws.

**Figure 8:**
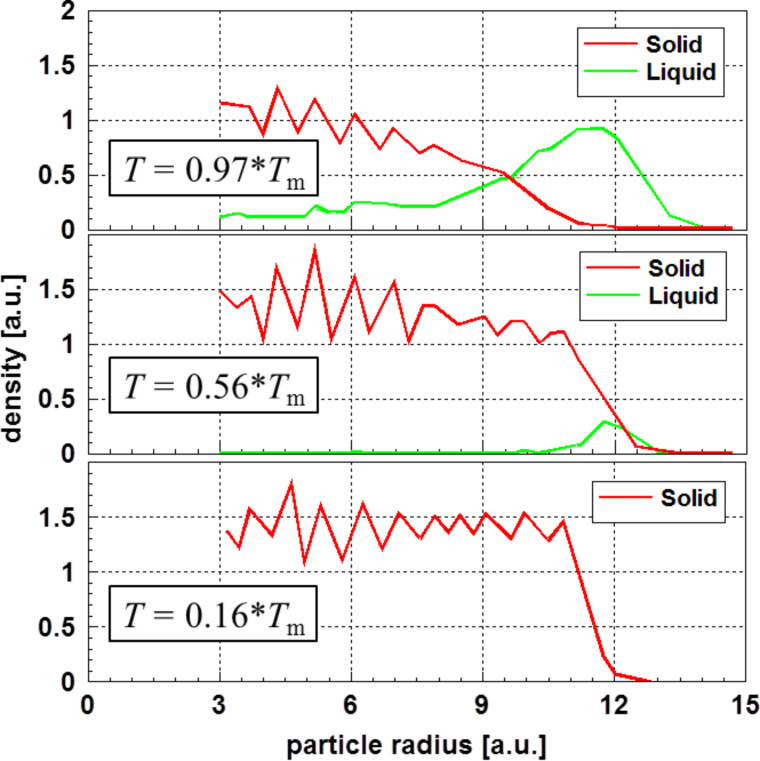
Radial profiles of the density for a fcc metal cluster consisting of 3302 atoms versus the particle radius [43]. One observes the formation of a noncrystalline layer at the surface, which is considered liquid-like in the vicinity of the melting temperature of the particle, *T* = 0.62·*T*_m_ (*T*_m_ – melting temperature of the bulk material).

Compared to continuum thermodynamics, molecular dynamic calculations give a deeper insight into the structure of small particles. A study on structure and melting of a particle with fcc structure composed of 3302 atoms (which is equivalent to a particle diameter *D* in the range of 6–7 nm; *D* = 23 atoms times an atomic diameter of 0.288 nm) was performed by Sang et al. [43]. Figure 8 displays some of these results showing radial profiles of the density as function of the particle radius and temperature. Density, radius, and temperature are given in arbitrary units.

Figure 8 demonstrates that the nanoparticle surface becomes liquid-like in the vicinity of the melting temperature. Therefore, one may assume a decreasing surface energy close to the melting point. Such a phenomenon may not be expected at low temperatures.

**Figure 9:**
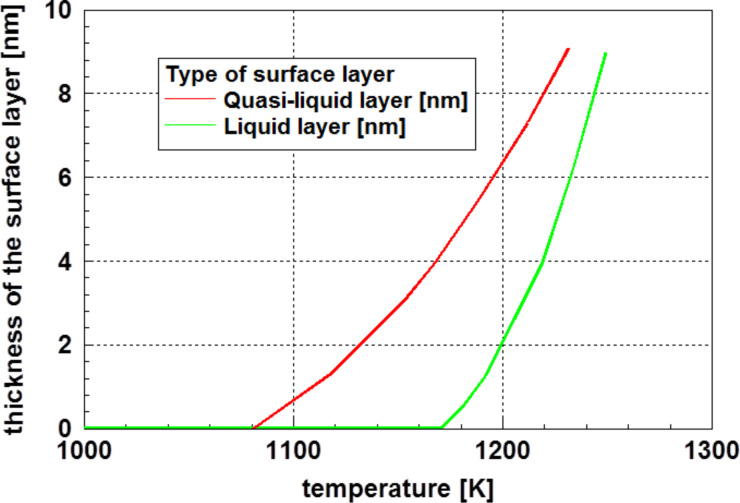
Thickness of the liquid and the quasi-liquid transition layer close to the surface of a 18 nm gold particle calculated by molecular dynamics [46]. The phenomenon of a layer with reduced order is restricted to temperatures not too much below the melting point of the particles. Adapted with permission from [46], copyright 2013 American Chemical Society.

In most cases, molecular dynamic simulations of the melting process of small nanoparticles show, at significantly lower temperatures compared to the melting point, a liquid or quasi-liquid surface [43–47]. Certainly, this finding promotes the expectation of a reduced surface energy. Results for silver nanoparticles, shown by Alarifi et al. [46], demonstrate the existence of both an outer liquid layer and subsequent a quasi-liquid transition layer at the surface of nanoparticles. Even when both the quasi-liquid and the liquid layer are not crystallized, they can be distinguished by their energy. Figure 9 displays the thickness of these two kinds of surface layers for 18 nm gold particles as function of the temperature. These layers, with reduced order, are observed (at least for silver particles) at temperatures not too much below the melting point.

**Figure 10:**
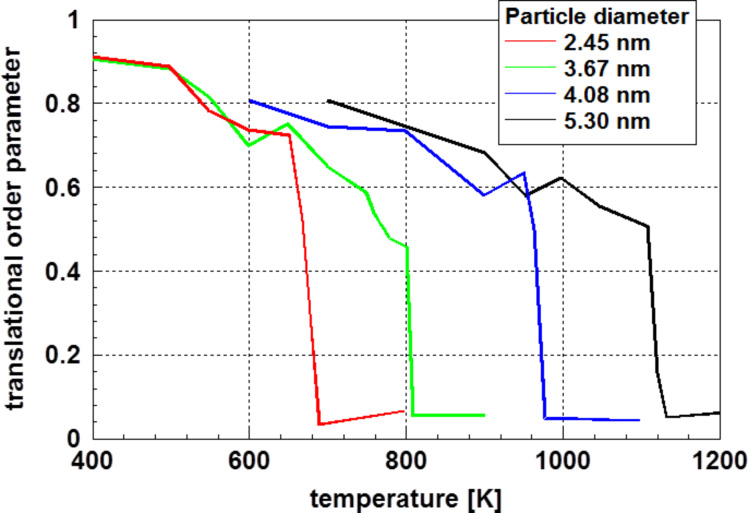
Translational order parameter for gold particles of different particle diameter as function of the temperature obtained by molecular dynamics simulations [47]. Even at a temperature significantly different from the melting temperature, perfect crystallization is not observed.

Molecular dynamics calculations using gold as an example resulted in a similar behavior. Qiao et al. [47] used, similarly as Chang and Johnson [35], an order parameter defined as the “translational order parameter” [48], which is 1 for perfectly crystallized materials and 0 for liquids, as a function of temperature and radius to characterize the status of crystallization. Figure 10 displays some of these results for four different particles sizes as function of the temperature [47].

The results displayed in Figure 10 shows an important feature: independent of the particle diameter, even at a temperature significantly lower than the melting temperature, the particles do not show perfect crystallization. It is important to keep in mind that the values given in Figure 10 are averaged over the whole particle. One may assume that a significant contribution to this reduced order stems from the surface-near zone of the particle. Consequently, these results support (at least in the temperature range shown in Figure 10) that the surface energy decreases with decreasing particle diameter caused by the reduced surface energy of the liquid state.

**Figure 11:**
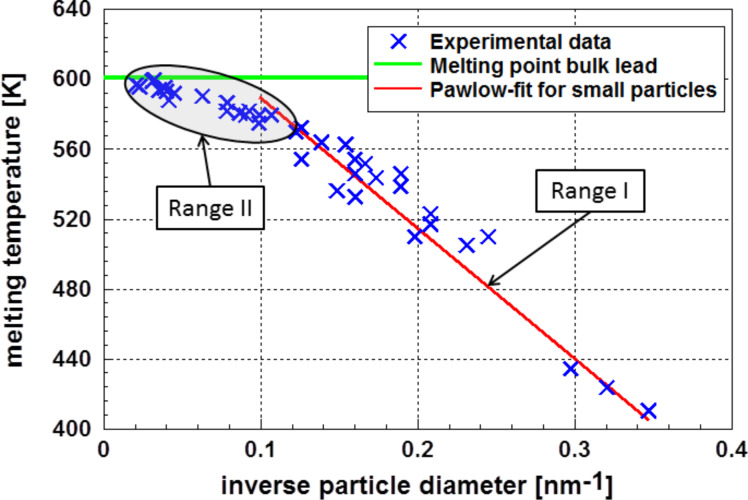
Melting temperature of lead according to Coombes [16]. This figure shows two ranges of melting temperatures dependent on the particle diameter. Range I follows the Pawlow relation; in this range the particles melt as a whole. In Range II the melting starts at the surface of the particle.

The formation of a liquid phase at the surface of lead particles was already experimentally observed by Coombes [16]. These results are depicted in Figure 11. In this figure, one clearly realizes two ranges of particle sizes: Range I – particles smaller than approximately 7.5 nm follow the Pawlow equation; Range II –larger particles, where melting starts at the surface, display a different behavior.

The results presented in Figures 8, 9, and 11 are not only consistent in interpretation, but are also observed experimentally.

Looking at the calculation of the surface energy, one is primarily confronted with the question of definition. Based on the general assumption describing the surface energy as excess energy [4], Medasani et al. [49] defined the total surface energy γ as the energy difference between *n* atoms in the bulk, *n*ε_bulk_ and the same number of atoms forming a nanoparticle with the surface *a*. Therefore, the surface energy γ may be defined as

[8]
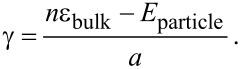


The quantity *E*_particle_ is the total energy of the nanoparticle. This approach should be a simple approach to interpreting the surface energy; it is highly applicable for many materials. However, it is well known that the structure (and therefore the binding energy of nanoparticles) is size-dependent [25]. Furthermore, in many cases, nanoparticles exhibit a highly symmetrical structure as compared to the bulk material [50]. Nanoparticles may even exhibit a quasi-liquid or glassy structure, states having no equivalents in the bulk [14]. Recent calculations of the surface energy of gold as function of the particle size by Ali et al. [51] have predicted an increase of the surface energy with decreasing particle size. However, these authors used the coordinates of the surface atoms to calculate the surface of the particle. Earlier, de Heer [52] pointed out that, looking at the size of a nanoparticle, one has to take care of the “electron spill-out”. De Heer proposed a correction of the particle radius of approximately 0.045–0.079 nm.

**Figure 12:**
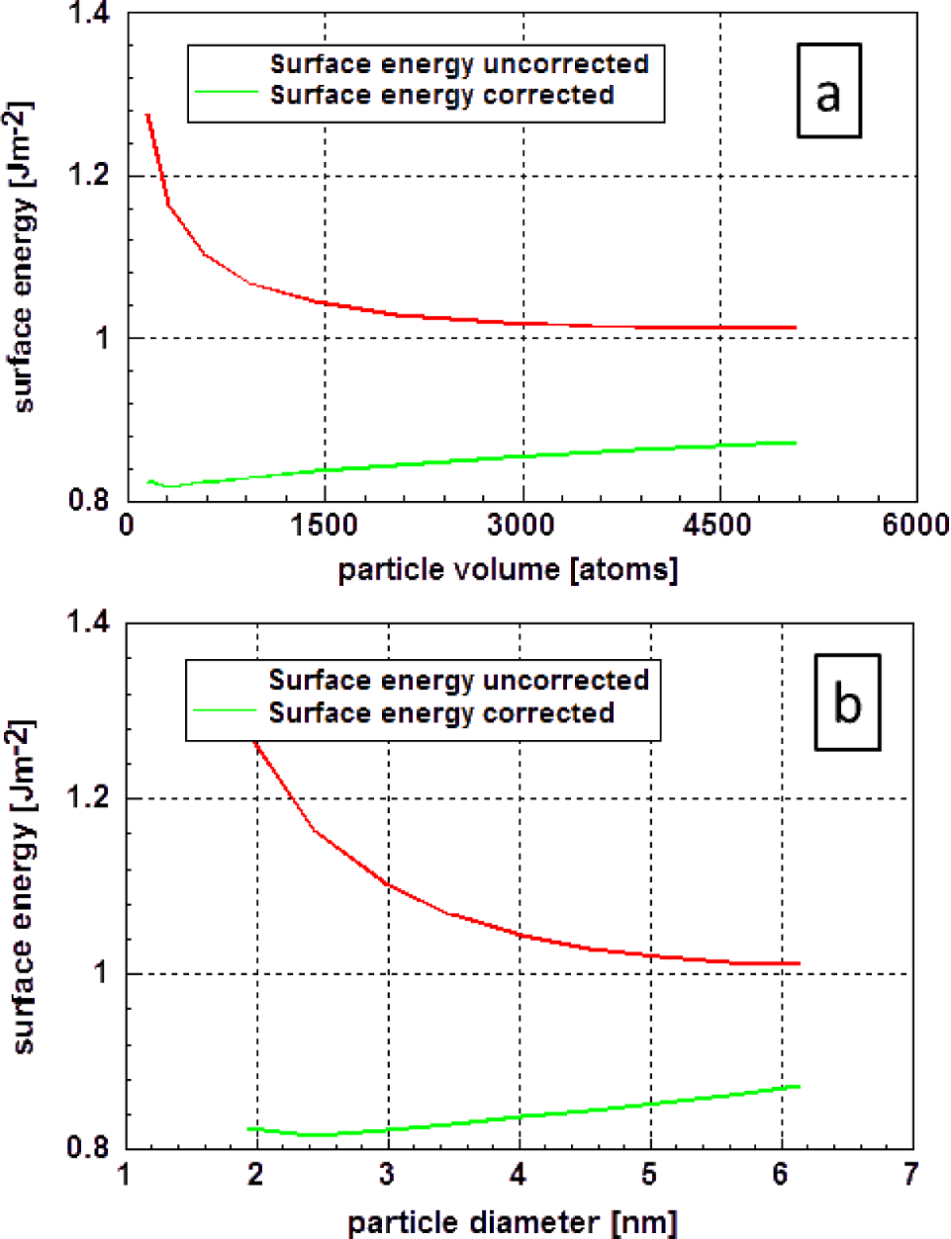
Surface energy of gold as function of the particle size according to Ali et al. [51]. The graphs show the original data of Ali et al. [51] and that corrected according to Holec et al. [53]. In the original paper, the size of the icosahedron-shaped particle is defined by the number of atoms in the particle (a). The size dependence of the surface energy, obtained by both assumptions, is plotted versus the particle diameter assuming a spherical particle of equal volume (b).

According to the results of a very detailed study of the electronic structure by Holec et al. [53], the value for the correction of the diameter should be in the range of an atomic diameter (≈0.288 nm). This correction is material dependent since its origin is the extent of the electronic cloud [54], which depends on the number of valence electrons, density of material (i.e., spatial density of atoms), etc. One also expects that this correction slightly increases with temperature (similar to the thermal expansion), however, this is unlikely to have any significant effect (and is expected to be marginal in comparison to other simplifications). Finally, no significant influence of the particle shape is expected as its origin (i.e., the spatial extent of the electronic cloud – because an atom is not a point-like object but rather a “sphere” of finite diameter) does not depend on the particle shape. Figure 12 displays the original data of Ali et al. [51] and the corrected data according to Holec et al. [53]. Ali et al. assumed highly crystalline icosahedral particles. In Figure 12, the surface energy is plotted versus both the particle volume (a) and the particle diameter (b). As an approximation, the particle diameter was calculated assuming spherical particles with equal volume. Interestingly, the corrected data show a slight decrease of the surface energy with decreasing particle size. Possibly, this is a result of the formation of a liquid-like layer at the surface.

**Figure 13:**
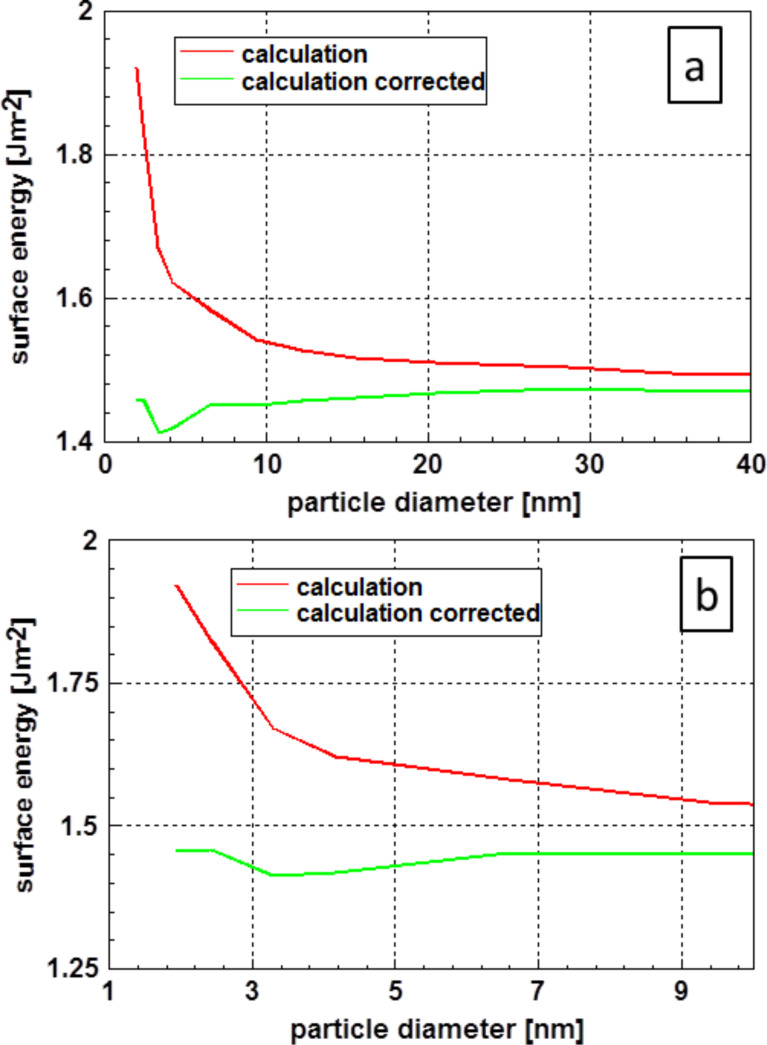
Results of molecular dynamic calculations of the surface energy of gold [54]. (a) All the results, whereas the range of small particles is displayed in more detail in (b). It is important to realize the increase of the surface energy that occurs after the minimum around the 3 nm particle diameter mark. The values corrected according to Holec et al. [53] take the physical size of the nanoparticles into account.

The results of the molecular dynamics calculations of Holec et al. [54] for the surface energy of spherical gold nanoparticles, depicted in Figure 13, are quite comparable with the values published by Ali et al. [51]. The particle surface in these calculations was also determined using the corrected particle diameter determined according to Holec et al. [53]. It is important to note that after the decrease of the surface energy down to a particle diameter of approximately 3 nm, an increase was found. This slight minimum is more visible in part (b) of this figure. As these data were obtained from fully relaxed crystalline material at a temperature 0 K, one may assume that the data are fully deterministic.

**Figure 14:**
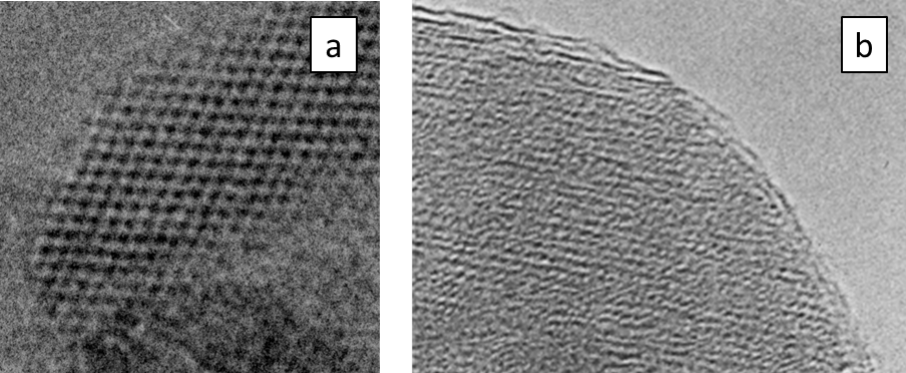
High-resolution electron micrograph of a zirconia, ZrO_2_, and an alumina, Al_2_O_3_ nanoparticles. (a) A perfectly (up to the surface) crystallized zirconia particle [55]. In contrast, the alumina particle displayed in (b) shows that the lattice fringes do not continue to the surface of the particle [56].

The results presented for metal particles are also found in a similar manner for ceramic particles. Typical examples are demonstrated using electron micrographs of ceramic particles. Figure 14 displays high-resolution electron micrographs of zirconia (a) and alumina (b) particles taken at room temperature [55–56]. The melting point of zirconia is significantly higher than that of alumina. In both cases, the temperature during synthesis was around 750 K. Therefore, considering the previous considerations, it is not astonishing that the zirconia particle is perfectly crystallized at the surface, whereas the alumina particle shows a noncrystalline layer at the surface.

**Figure 15:**
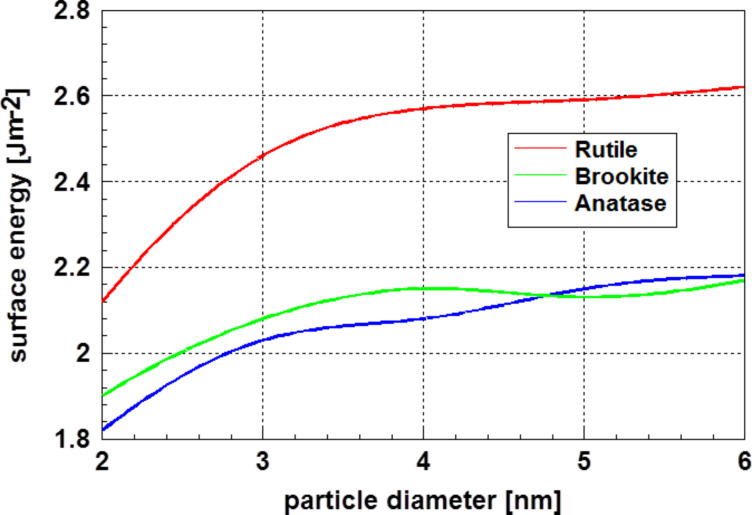
Surface energy of the three modifications of titania at a temperature of 300 K as function of the particle diameter according to Naicker et al. [57]. The reduction of the surface energy with decreasing particle diameter is remarkable. Adapted with permission from [57], copyright 2005 by America Chemical Society.

Studies devoted to surface energy of ceramic materials based on molecular dynamics calculations are quite rare. Naicker et al. [57] calculated the surface energy of three modifications of titania, TiO_2_, as a function of the particle size. For these three modifications, the calculations resulted in a significant decrease of the surface energy with decreasing particle diameter. Within this study, the geometry of the particles was determined from the coordinates of the atoms obtained by molecular dynamics. A special correction for the electron cloud was not reported. Figure 15 displays the result obtained at a temperature of 300 K. These results are in accordance with similar results obtained for metal particles and correlate with the high-resolution electron micrographs displayed in Figure 14.

#### Ab initio calculations

Ab initio calculations are based on solutions of the Schrödinger equation. However, it is well known that, except for the hydrogen atom, exact solutions of this equation do not exist. Therefore, a large number of methods for numerical solutions have been developed.

Most successful are calculations based on density functional theory (DFT). This kind of modelling has some restrictions, i.e., the availability of a supercomputer and the limitation of the temperature to 0 K. To some extent, the latter limitation and also the restriction to particles with a relatively small number of atoms can be overcome by combining ab initio calculations with molecular dynamics simulations.

**Figure 16:**
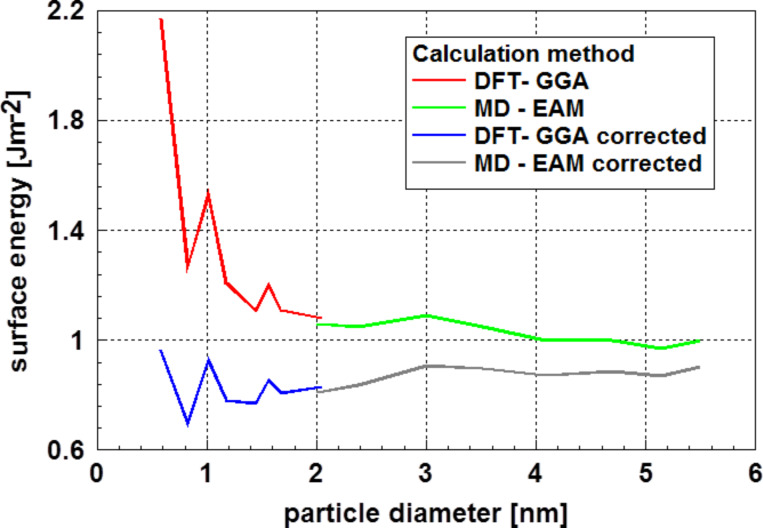
Surface energy of silver particles as function of the particle diameter [49]. This graph shows the original values and those corrected according to Holec et al. [53]. Except the systematic variation in the values for particles with sizes below ca. 2 nm, it is remarkable that there is only a relatively small influence of the particle diameter on the surface energy.

Typical examples for the application of this computational strategy can be found in papers from Medasani et al. [49,58–59]. Figure 16 displays the results for calculations of the surface energy of silver particles [49,58]. (The abbreviation DFT-GGA stands for density functional theory using a generalized gradient approximation, and EAM for an empirical embedded atom method.) The values for the surface energy were calculated using Equation 8. Medasani et al. [49,58–59] showed the influence of a correction for the particle size according to de Heer [52]. This correction results in an increase in the particle radius from 0.045 nm to 0.079 nm. According to the results of a very detailed study of the electronic structure by Holec et al. [53] the value for the correction of the diameter should be in the range of an atomic diameter (≈0.288 nm). Figure 16 displays the original values of the surface energy and that after correction according to Holec et al. [53]. The calculations by Medasani et al. are based on the assumption of crystallized particles.

Looking at the results, it is remarkable that the values for particles with a diameter smaller than 2 nm (obtained by DFT calculations) vary so drastically. Furthermore, it is interesting to note that after the correction, the dependence of the surface energy on the diameter is more or less in the range of the dataset as a whole. This result is somewhat similar to that obtained for gold as displayed in Figure 13.

**Figure 17:**
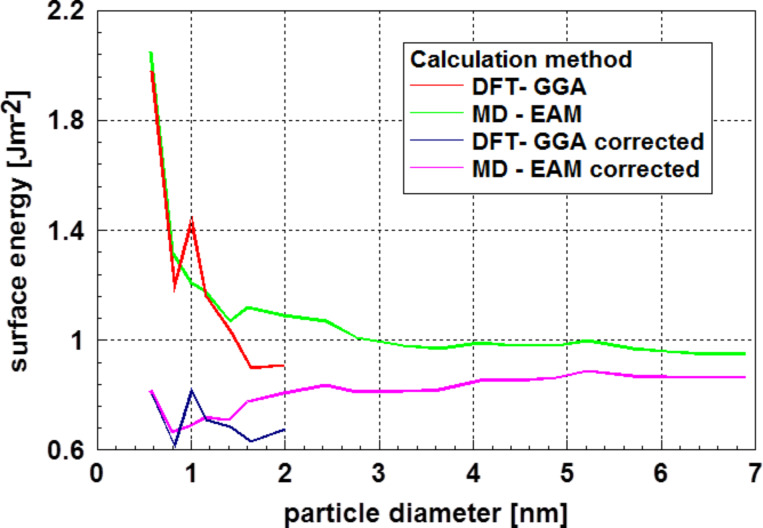
Surface energy of aluminum particles as function of the particle diameter [58]. This graph shows both the original values and those corrected according to Holec et al. [53]. With the exception of the systematic variation of the values for particle diameters below 2 nm, the corrected values show a trend of decreasing surface energy with decreasing particle size for particle diameters larger than 2 nm.

The results for aluminum particles displayed in Figure 17 obtained by Medasani et al. [58] are, in general, very similar. Also, in this case, the particles were assumed to be crystalline and their shape close to spherical. After correction, a very moderate dependence of the surface energy on the particle diameter is obtained in this case. Only a slight decrease with decreasing particle size is observed. It is remarkable that for particles of diameter less than ≈2 nm, the results vary in a pattern similar to the case of silver particles. Interestingly, the extrema are found at the same particle diameters in both cases. One may question if this is a systematic error or an indication of a structural feature. However, it is remarkable that the first extremum occurs, in both cases, for a cluster consisting of 13 atoms and the second one for a cluster size of 43 atoms. The number 13 is a so-called “magic number” for cluster sizes; however, the number 43 is far off from the second magic number for clusters consisting of 55 atoms.

More detailed insight is provided by a thorough ab initio study analyzing the Au_55_ cluster performed by Holec et al. [53] and Vollath et al. [14]. These studies established the fact that the most stable state of this cluster is glassy. The accompanying determination of the surface energy using the approach of Medasani et al. [49] resulted in a value of 1.36 J m^−2^ [51]. Since there are some doubts using this approach, a new concept for estimation of the surface energy, based on the Kelvin equation, was developed. Extrapolating the Kelvin equation to 0 K, one obtains

[9]
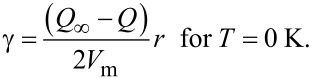


**Figure 18:**
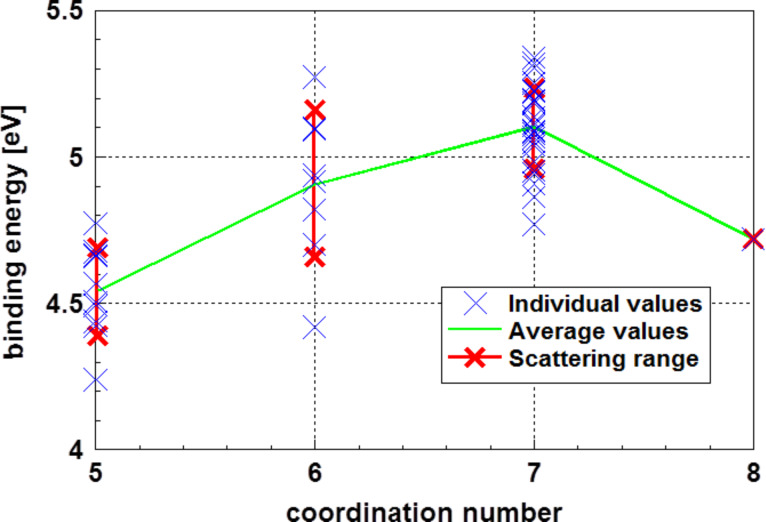
Binding energy of the atoms in the outmost layer of an Au_55_ cluster [14]. Additionally, for each coordination number, the 1σ scattering range is indicated. The atoms with the highest probability to be evaporated are those with the smallest coordination number and the smallest binding energy.

In this equation the quantities *Q*_∞_ and *Q* stand for the enthalpy of sublimation of the bulk and the enthalpy of sublimation for the particle in question, respectively. *V*_m_ stands for the molar volume of the atoms in the particle and *r* for its radius. Also in this case, the definition of the particle radius is a crucial problem that may be solved using a correction derived by Holec et al. [53]. The enthalpy of sublimation is determined as the binding energy of the outmost atoms of the particle. Figure 18 displays the binding energy for the atoms of the outmost layer of the glassy Au_55_ cluster. To estimate the surface energy, the binding energy of the atoms with the least coordination number must be selected. Since this is, to some extent, a random process, the scatter of these binding energies gives an indication for the scattering of the result. These considerations led to a value of 1.51 ± 0.68 J m^−1^ for the surface energy of the Au_55_ cluster at 0 K, also covering the value of the surface energy calculated according Equation 8.

Comparing the two approaches to calculate the surface energy shown above, one realizes a fundamental difference: The most common approach [4] (applied by Medasani et al. [49]) does not take the vapor pressure of the material into account. The approach developed by Vollath et al. [14] is based on the Kelvin equation and assumes, therefore, equilibrium with the vapor phase. However, at 0 K, this difference is not relevant.

## Conclusion

Studying the size dependence of the surface energy of nanoparticles, one finds different tendencies depending on the method of calculation. Considerations based on continuum thermodynamics predict decreasing values of the surface energy as function of decreasing particle size. This tendency is supported by

- the reduction of the number of next neighbors of the surface atoms, although this tendency is at least partly compensated by an increase of the binding energy and
- by the formation of a quasi-liquid phase at the surface.

The latter finding has been confirmed by experimental results. However, these results have not been reflected in the published values for the surface energy. On the contrary, the molecular dynamics calculation led to significantly increasing values of the surface energy with decreasing particle size. However, it was shown that the predicted increase of the surface energy may be explained by the insufficient determination of the particle diameter and, consequently, of the particle surface. After applying improved values for the particle size and shape, the resulting values of the surface energy no longer exhibit a significant dependence on the particle size.

The results of the ab initio calculations show a similar tendency. Also, in this case, the values for the surface energy show only a minor dependence on the particle size after applying the correction of the particle size according to Holec et al. [53]. However, in the size range below ≈2 nm, a “systematic scattering” or variation of the results is observed, which could be explained by structural variations or systematic errors in the calculations. The argument of structural variations is supported by findings of Vollath et al. [14] that the Au_55_ cluster is not crystallized but rather glassy. Such structural variations were, on the basis of continuum thermodynamics, considerations predicted by Vollath et al. [24], too.

Summarizing the various results for the surface energy study of nanoparticles, one may state that

- there exists only a minor dependence of the surface energy on the particle size,
- at higher temperatures, where the surface is covered with a liquid or quasi-liquid layer, a slight decrease of the surface energy may be expected, and
- indications exist that structural modifications for particles of diameter less than ≈2 nm may lead to a minor scattering of the values for the surface energy.

Summarizing these findings, it may be recommended to use data for the bulk material when data for the equivalent nanomaterial is not available.
